# Multi-omics analysis reveals the prognostic and tumor micro-environmental value of lumican in multiple cancer types

**DOI:** 10.3389/fmolb.2023.1158747

**Published:** 2023-08-24

**Authors:** Zehuai Guo, Zeyun Li, Ming Chen, Xiangjun Qi, Zhe Sun, Siqi Wu, Xuenan Hou, Mengli Qiu, Yang Cao

**Affiliations:** ^1^ Department of Internal Medicine, Cancer Hospital of Shantou University Medical College, Shantou, China; ^2^ Guangzhou Huaxia Vocational College, Guangzhou, China; ^3^ Shenzhen Hospital (Futian) of Guangzhou University of Chinese Medicine, Shenzhen, China; ^4^ The First Clinical School of Guangzhou University of Chinese Medicine, Guangzhou, China; ^5^ The First Affiliated Hospital of Guangzhou University of Chinese Medicine, Guangzhou, China

**Keywords:** lumican, pan-cancer, tumor microenvironment, cancer-associated fibroblasts, immunotherapy, multi-omics

## Abstract

**Background:** Lumican (LUM), a proteoglycan of the extracellular matrix, has been reported to be involved in the regulation of immune escape processes, but the data supporting this phenomenon are not sufficient. In this study, we aimed to explore the links among LUM expression, survival, tumor microenvironment (TME), and immunotherapy in 33 cancer types.

**Methods:** Data from several databases, such as UCSC Xena, GTEx, UALCAN, HPA, GEPIA2, TISIDB, PrognoScan, TIMER2, and GEO, as well as published studies, were used to determine the relationship between LUM expression and clinical features, TME, heterogeneity, and tumor stemness.

**Results:** The expression of LUM was statistically different in most tumors versus normal tissues, both at the RNA and protein expression levels. High expression of LUM was typically associated with a poor prognosis in tumors. Additionally, immune scores, six immune cells, four immunosuppressive cells, cancer-associated fibroblasts (CAFs)-associated and immunosuppressive factors, tumor mutation burden (TMB), microsatellite instability (MSI), DNAss, and RNAss were all significantly associated with LUM. Among them, LUM expression displayed a significant positive correlation with CAFs and their factors, and exhibited immunosuppressive effects in six independent immunotherapy cohorts.

**Conclusion:** Multi-omics analysis suggests that LUM may have been a prognostic marker, contributed to immunosuppression in the TME, and decreased the effectiveness of immune checkpoint inhibitors.

## 1 Introduction

The tumor microenvironment (TME), as the environment for tumor cell survival and material basis for influencing tumor development, is mainly composed of non-tumor cells (such as immune cells, cancer-associated fibroblasts (CAFs), vascular endothelial cells), extracellular matrix (ECM), and various cytokines (Arneth, 2019). The extracellular matrix provides the physical scaffold for tumor cell motility, adhesion, and metastasis (Nikitovic et al., 2014). As ECM components, small leucine-rich proteoglycans (SLRPs) are connected with cell receptors, which can greatly influence cancer progression and cancer cell proliferation, either by promoting it or inhibiting it (Chen and Birk, 2013; Solis-Hernandez et al., 2021). Lumican (LUM) is a class II SLPRs, consisting of C-terminal regions containing cysteine residues, N-terminal regions containing signal peptides and tyrosine sulfate, LRR, and a 16 amino acid peptide (Giatagana et al., 2021). As an important part of the human body, ECM exerts various effects on cell shape, function, migration, proliferation, as well as differentiation via signal transduction systems (Pickup et al., 2014). LUM is strongly expressed in the cornea and skin. In these organs, LUM deficiency leads to decreased corneal transparency (Quantock et al., 2001; Beecher et al., 2006) and poor healing abilities of skin wounds (Karamanou et al., 2018).

Biologically, LUM inhibits or enhances cancer cell growth, differentiation, migration or metastasis by regulating growth receptors and signaling pathways (Giatagana et al., 2021). In gastric cancer, overexpressed LUM increases the risks of poor prognostic outcomes by activating 14 signaling pathways (Chen et al., 2020). Moreover, in colon cancer (Seya et al., 2006), liver cancer (Mu et al., 2018), neuroblastoma (Wang et al., 2017), osteosarcoma (Nikitovic et al., 2011), and chondrosarcoma (Papoutsidakis et al., 2020) among others, overexpressed LUM has been associated with worse clinical outcomes. In melanoma, elevated LUM levels inhibit cancer cell proliferations by suppressing MMP-14 activities or inhibiting focal adhesion kinase phosphorylation (Karamanou et al., 2021). Besides, LUM exhibits antitumor effects in lung cancer (Yang et al., 2018), breast cancer (Karamanou et al., 2020) and pancreatic ductal adenocarcinoma (Li et al., 2019). MMP-9, mitogen activated protein kinases (MAPK) and Focal adhesion kinases (FAK) are activated in LUM-mediated pro-tumorigenic actions. The anti-tumorigenic potential of LUM is combined with epithelial-mesenchymal transition (EMT) and downregulation of MMP-14, extracellular signal-regulated kinases (ERK), and FAK mediated pathways to inhibit carcinogenesis (Appunni et al., 2021). It has been reported that LUM affects inflammatory as well as immune responses and may be a potential tumor-associated inflammatory modulator (Nikitovic et al., 2014). On the other hand, LUM enhances cancer cell sensitivity to chemotherapeutic agents by inhibiting cell metabolism (Li et al., 2016). With regards to immune responses, LUM is involved in immune escape in the colon cancer microenvironment (Zang et al., 2021). In conclusion, LUM is a potential therapeutic target for cancer (Karamanou et al., 2020).

In conclusion, LUM has different roles in different tumors, and this heterogeneity seriously affects our overall understanding of LUM, so this study performed a pan-cancer analysis of LUM to explore the overall trend of LUM. In the study, we comprehensively analyzed the expressions and prognostic significance of LUM and explored the relationship between LUM with major TME components and immunotherapy to reveal its regulatory mechanisms in 33 tumors. Our findings elucidate on the significance of LUM in cancer therapy.

## 2 Materials and methods

### 2.1 Data collection

The FPKM value of gene expressions, somatic mutation data, and clinicopathological information for 33 human cancers were downloaded from UCSC Xena (https://xenabrowser.net/datapages/). Full names and abbreviations for the 33 cancers are listed in Table 1. Normal tissue data for LUM expressions were downloaded from the GTEx website (https://www.gtexportal.org/). To assess the relationship between LUM expressions and the efficacy of immune checkpoint inhibitor therapy, we searched for relevant study cohorts involving immune checkpoint inhibitor therapy for which complete clinical and gene expression information had been published. IMvigor210, a very classical research project, is a cohort study of the biological phenotype of patients with metastatic uroepithelial cancer treated with anti-PD-L1 drugs (Atezolizumab) (Mariathasan et al., 2018). The GSE78220 cohort for studying Pembrolizumab in melanomas and the GSE67501 cohort for studying Nivolumab in renal cell carcinoma were downloaded from the GEO Database (https://www.ncbi.nlm.nih.gov/geo/).

**TABLE 1:** The 33 tumors analyzed in this study and their sample sizes.

Full names	Abbreviations	N
Adrenocortical carcinoma	ACC	79
Bladder urothelial carcinoma	BLCA	408
Breast invasive carcinoma	BRCA	1,098
Cervical squamous cell carcinoma and endocervical adenocarcinoma	CESC	306
Cholangiocarcinoma	CHOL	36
Colon adenocarcinoma	COAD	458
Lymphoid neoplasm diffuse large B-cell lymphoma	DLBC	48
Esophageal carcinoma	ESCA	162
Glioblastoma multiforme	GBM	167
Head and neck squamous cell carcinoma	HNSC	502
Kidney chromophobe	KICH	65
Kidney renal clear cell carcinoma	KIRC	531
Kidney renal papillary cell carcinoma	KIRP	289
Acute myeloid leukemia	LAML	151
Brain lower grade glioma	LGG	525
Liver hepatocellular carcinoma	LIHC	373
Lung adenocarcinoma	LUAD	515
Lung squamous cell carcinoma	LUSC	501
Mesothelioma	MESO	86
Ovarian serous cystadenocarcinoma	OV	379
Pancreatic adenocarcinoma	PAAD	178
Pheochromocytoma and paraganglioma	PCPG	183
Prostate adenocarcinoma	PRAD	496
Rectum adenocarcinoma	READ	167
Sarcoma	SARC	263
Skin cutaneous melanoma	SKCM	471
Stomach adenocarcinoma	STAD	375
Testicular germ cell tumors	TGCT	156
Thyroid carcinoma	THCA	510
Thymoma	THYM	119
Uterine corpus endometrial carcinoma	UCEC	544
Uterine carcinosarcoma	UCS	56
Uveal Melanoma	UVM	80

### 2.2 Expression characterization of LUM in tumor tissues

We extracted the gene expression data from the TCGA and GTEx databases separately using a perl script, and used the limma package and its normalizeBetweenArrays algorithm to merge and normalize the gene expression data from the two databases. The Wilcoxon rank sum test was used to evaluate the difference in LUM expression between a tumor group and a normal group, as well as among different stages of tumor groups. The GEPIA2 (http://gepia2.cancer-pku.cn) and TISIDB (http://cis.hku.hk/TISIDB) databases were used to investigate the relationship between LUM expression and the stage of the pan-cancer. The CPTAC database in the UALCAN portal website (http://ualcan.path.uab.edu/index.html) was used to investigate differences in LUM protein expressions between normal and tumor tissues (Chandrashekar et al., 2022). Regrettably, only 10 tumor types (BRCA, COAD, GBM, HNSC, KIRC, LIHC, LUAD, OV, PAAD, and UCEC) had their LUM protein data available for analysis. To further investigate the differential expression of LUM at the protein level, we acquired immunohistochemical images of 10 different types of tumor tissues and their corresponding normal tissues from HPA (https://www.proteinatlas.org).

Subsequently, genetic alterations of LUM in the TCGA pan-cancer atlas cohort of cBioPortal (http://www.cbioportal.org/) were visualized. Through the “View 3D Structure” of the “Mutations” module, the most frequent mutation sites of LUM were displayed in the 3D schematic diagram of the protein structure of LUM.

### 2.3 Survival analysis of LUM expression levels

Data for survival time of cancer patients downloaded from UCSC Xena website included Overall Survival (OS), Disease Free Survival (DFS), Progression Free Survival (PFS), and Disease-Specific Survival (DSS). Patients with different tumour types were classified into high and low LUM expression groups based on median LUM expression values. Using “survival” and “survminer” packages in R, univariate Cox regression and Kaplan-Meier (KM) analyses were performed to determine the correlation between LUM and four survival indicators in 33 cancers. Next, we designed a forest map using “ForestPlot” in R software and Adobe Photoshop 2021 software. The PrognoScan database (http://dna00.bio.kyutech.ac.jp/PrognoScan/index.html) was used to assess the relationship between LUM expression and patient survival outcomes, and meta-analysis was used to further confirm the association between LUM expression and breast cancer survival.

### 2.4 Correlations between LUM expressions and TME in pan-cancers

We used the ESTIMATE algorithms in “ESTIMATE” and “limma” packages in R to calculate stromal and immune scores for each patient. Then, we evaluated the correlations between LUM expressions with stromal and immune scores using “ggExtra”, “ggplot2” and “ggpubr” in R.

Then, the TIMER2 website (http://timer.cistrome.org/) was used to analyze correlations between LUM expressions and 6 immune cell types (including B cells, CD4^+^ T cells, CD8^+^ T cells, dendritic cells, macrophages, and neutrophils) and 4 kinds of immunosuppressive cells, including CAFs, regulatory T cells (Tregs), myeloid-derived suppressor cells (MDSCs), and M2 subtype of tumor-associated macrophages (M2-TAMs). Since the Timer2 website records many algorithms on immune cells, which are not conducive for visualization of the immune infiltrating landscape, results of the TIMER algorithm were visualized using “reshape2” and “RColorBrewer” R packages.

To validate the relationship between LUM and tumor immunosuppression, correlation analysis between LUM with CAFs-associated and immunosuppressive factors were evaluated. These factors were summarized based on previous studies (Lakins et al., 2018; Mao et al., 2021; Ding et al., 2022).

A high tumor mutation burden (TMB) and microsatellite instability (MSI) imply a good efficacy of immune checkpoint inhibitors. The TMB values were calculated from somatic mutation data from 33 cancers based on Perl scripts, while the MSI data were obtained from previous studies (Bonneville et al., 2017). Finally, “fmsb” in R was used to design radar diagrams to visualize the relationship between LUM and TMB/MSI.

What’s more, we obtained tumor stemness scores from previous studies for all TCGA tumor types (Malta et al., 2018), including RNA stemness scores (RNAss) and DNA stemness scores (DNAss), and subsequently integrated the tumor stemness scores with gene expression data to obtain a database by filtering samples with expression levels of 0. The Pearson correlation analysis was utilized to investigate the relationship between LUM expression and RNAss/DNAss.

Lastly, gene set enrichment analysis (GSEA) was performed to divide the samples into two groups based on LUM expression levels and retrieve statistically different pathways between the two groups from the subset based on c2.cp.kegg.v7.4.symbols.gmt.

### 2.5 Analysis of LUM expressions in predicting chemotherapeutic and immunotherapeutic efficacies

Associations between drugs and LUM expressions in the GDSC database were determined using the GSCA website (http://bioinfo.life.hust.edu.cn/GSCA/#/drug). Thirty of the most sensitive drugs were identified. Positive correlations meant that the LUM gene was drug resistant. Then, we verified the correlations between LUM transcriptome levels and chemotherapeutic responses in patients with OV, colorectal cancer, GBM and breast cancer on ROC Plotter Server (http://www.rocplot.org/site/index).

To elucidate on the prognostic significance of LUM after immunotherapy, the three retrieved immunotherapeutic studies were assigned into response and non-response groups, respectively. The response group had patients who achieved a complete response (CR) or partial response (PR) after immunotherapy, while the non-response group had patients with progressive disease (PD) or stable disease (SD). Differences in LUM expressions between non-response and response groups were determined by the Wilcoxon test. Finally, to investigate the cause of LUM-mediated immunotherapeutic insensitivity, correlations between LUM expressions and survival risk and cytotoxic T lymphocytes (CTL) in different cohorts with immunotherapy were queried in the “Query Gene” module of the TIDE website (http://tide.dfci.harvard.edu/).

## 3 Results

### 3.1 Features of LUM expressions in tumor tissues

**FIGURE 1:**
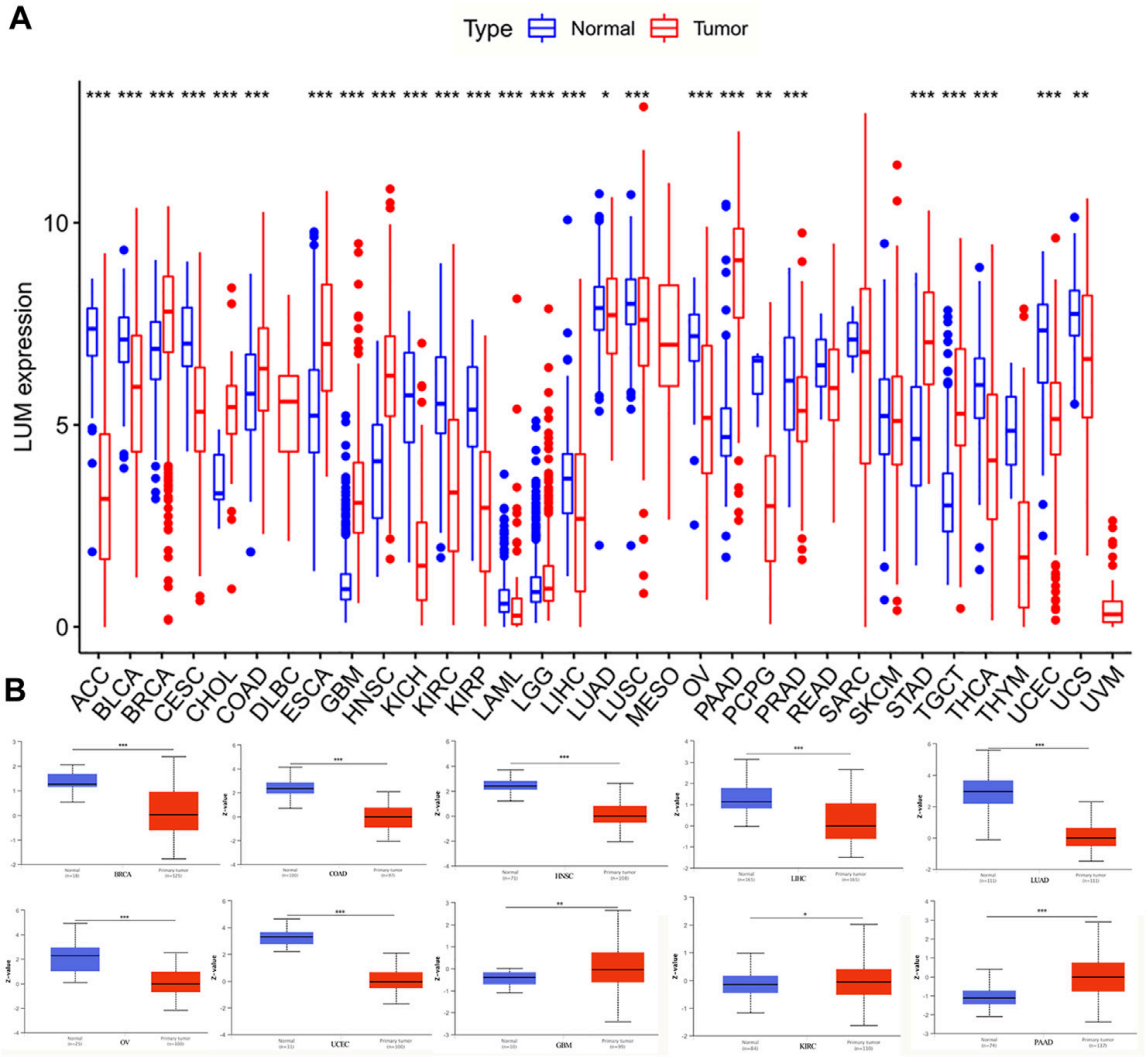
Differences in RNA and protein expression levels of LUM in different tumors. **(A)** Differential expression of LUM mRNA between tumor group and normal group in TCGA and GTEx databases. **(B)** Differential expression of LUM protein between normal tissue and BRCA, COAD, GBM, HNSC, KIRC, LIHC, LUAD, OV, PAAD and UCEC tissues. Z-values represent standard deviations from the median across samples for the given cancer type. Log2 Spectral count ratio values from CPTAC were first normalized within each sample profile, then normalized across samples. (*p < 0.05, **p < 0.01, ***p < 0.001).

After integrating the GTEx and TCGA databases, we compared differences in expressions of LUM between normal and tumor samples. Compared to normal tissues, LUM was differentially expressed in 26 of 33 tumors, among which the expressions of BRCA, CHOL, COAD, ESCA, GBM, HNSC, LGG, PAAD, STAD, and TGCT were higher, while those of ACC, BLCA, CESC, KICH, KIRC, KIRP, LAML, LIHC, LUAD, LUSC, OV, PCPG, PRAD, THCA, UCEC, and UCS were significantly lower (Figure 1A). To assess the differences in LUM gene at the translational level, the CPTAC database was used to compare differences in LUM protein levels between normal and tumor groups on the UALCAN website. Compared to GBM, LIHC, LUAD, OV, PAAD, and UCEC, variations in LUM protein levels in BRCA, COAD, HNSC, and KIRC were not in tandem with LUM RNA expressions (Figure 1B).

**FIGURE 2:**
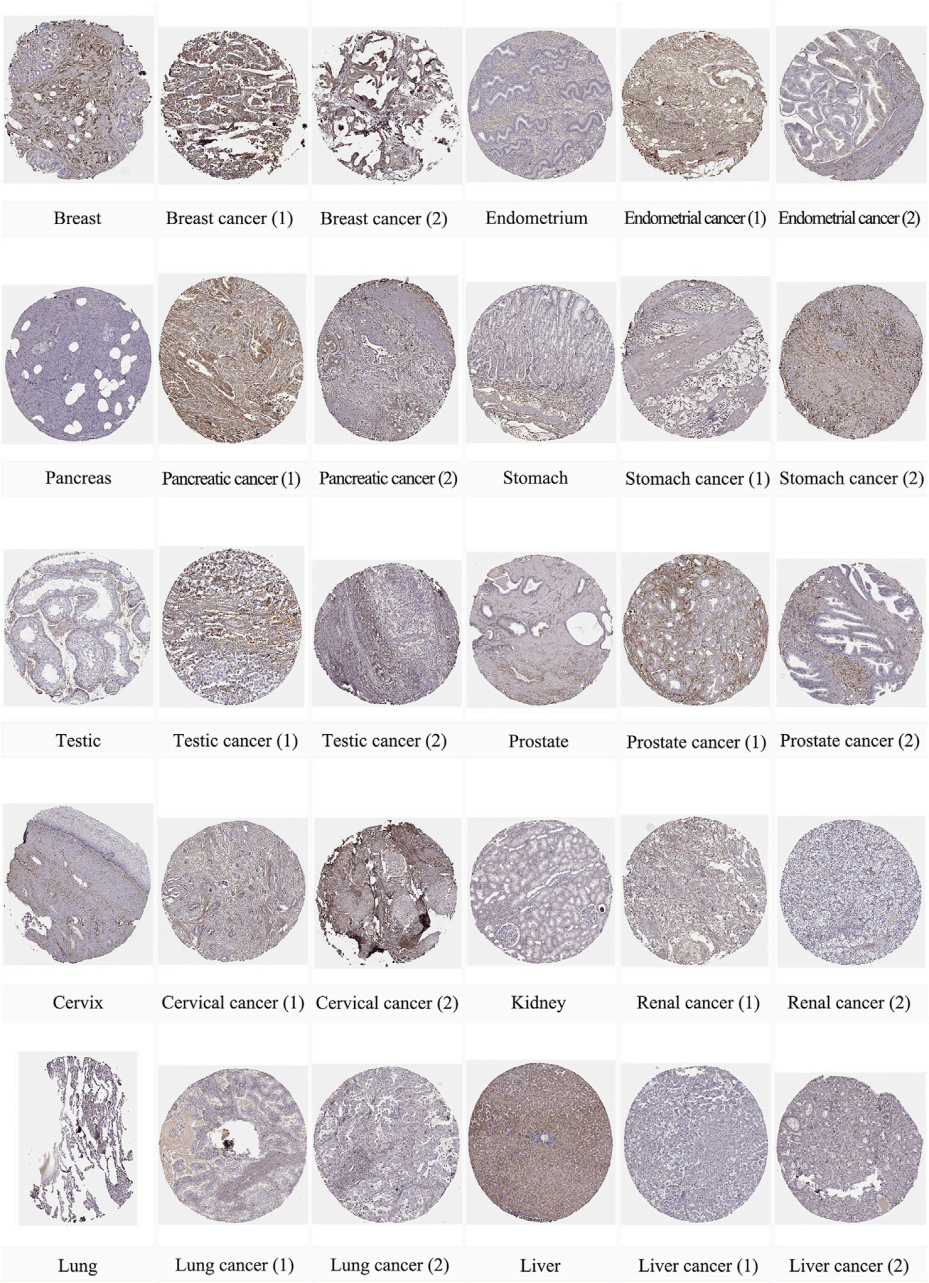
The distribution of LUM proteins in immunohistochemical images of normal and tumor tissues, including breast, endometrium, pancreas, stomach, testis, prostate, cervix, kidney, lung, and liver, as well as their malignant tissues.

In addition, we used immunohistochemical images from the HPA database to observe the location and expression levels of LUM protein expression in tumor tissues and normal organ tissues. As shown in Figure 2, significant staining of the extracellular matrix was observed in most tumor tissues. Additional cytoplasmic positivity of varying intensities was also shown in some cases of endometrial, pancreatic, breast, gastric, prostate, cervical, and renal cancers. Therefore, LUM protein expression in these tumor tissues may be higher than LUM expression in normal tissues. Notably, it was evident from the immunohistochemical images that LUM expression in hepatocellular carcinoma was lower than that in normal liver tissue, and LUM protein expression in liver tissue was mainly concentrated in hepatocytes rather than cholangiocytes.

**FIGURE 3:**
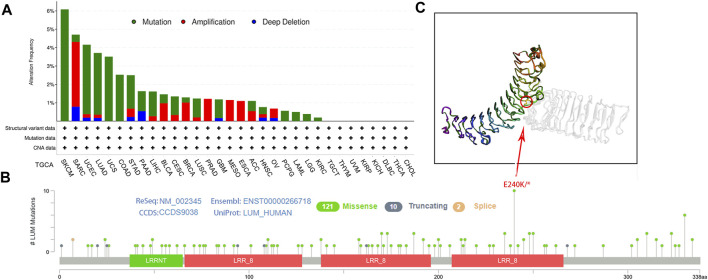
Characteristics of LUM expression in pan-cancer from the TCGA database as determined using the cBioPortal platform. **(A)** the proportion of various alteration types of LUM in different tumors. **(B–C)** the lollipop diagram showing the mutated site of LUM in pan-cancer and the most frequently mutated site in the 3D structure of LUM.

Then, genetic alterations of LUM in different tumors from the TCGA cohort were evaluated. In cBioPortal, most of the tumors exhibited a “mutant” phenotype as the main variant type, apart from SARC, BLCA, BRCA, PRAD, MESO, ESCA, ACC, and OV. Among them, SKCM was the tumor with the highest LUM mutation frequency, with 6.08%, while 3.53% of SARC patients had LUM gene amplification (Figure 3A). Next, we identified that the missense mutation of LUM was the primary type of genetic alteration, and the E240K/* alteration was detected in three cases of SKCM, two cases of COAD, and one case of BRCA, LUAD, STAD, UCEC and Rectal Adenocarcinoma (Figures 3B, C).

After dividing the tumors into four groups based on pathological stages, LUM expression was found to be statistically different in the stages of ACC, BLCA, ESCA, KIRC, KIRP, OV, STAD, THCA, THYM, and UCEC (Supplementary Figure S1A). Combined with the results of GEPIA2 and TISIDB analyses (Supplementary Figures S1B–S3), a more reliable conclusion can be drawn that LUM expression has a close relationship with the staging of BLCA, ESCA, KIRC, OV, and THCA. Despite the fact that LUM expression values decreased in some tumor stages, from an overall point of view, the expression of LUM showed an increasing trend with the tumor stage.

### 3.2 Prognostic significance of LUM across cancers in pan-cancer

**FIGURE 4:**
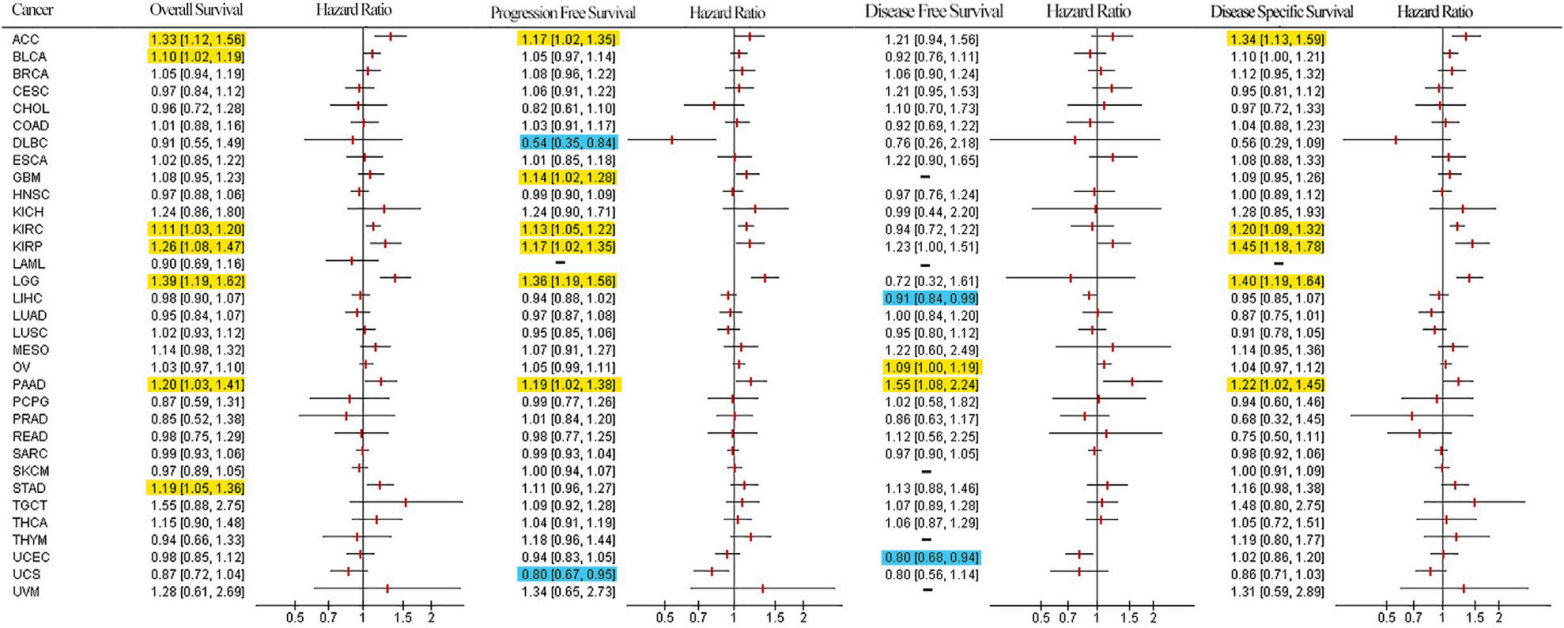
Survival forest plot based on univariate Cox regression analysis. Items highlighted in yellow indicate that LUM expression was negatively correlated with the survival indicator and items highlighted in blue indicate that LUM expression was positively correlated with the survival indicator (*p* < 0.05).

To determine the prognostic value of LUM, Cox and KM analyses were performed to assess correlations between LUM expressions and patient survival indices. Forest plots in Figure 4 show that elevated LUM expressions are predictors for poor OS in ACC, BLCA, KIRC, KIRP, LGG, PAAD, and STAD, poor PFS in ACC, GBM, KIRC, KIRP, LGG, and PAAD, poor DFS in OV and PAAD, and poor DSS in ACC, KIRC, KIRP, PAAD, and LGG. In contrast, PFS was longer in patients with high LUM expressions, compared to those with low LUM expressions in DLBC and UCS. Regarding DFS, elevated LUM levels were established to be beneficial in LIHC and UCEC. KM analysis revealed that elevated LUM expressions were associated with poor OS in ACC, KIRC, KIRP, STAD, and TGCT, poor DFS in ESCA, poor PFS in LGG, and poor DSS in ACC, KIRC, and KIRP. However, the DLBC patient with elevated LUM expressions had longer PFS and DSS (Supplementary Figure S4).

To further validate the prognostic value of LUM in pan-cancer, we performed survival analysis on multiple datasets using the PrognoScan database. The results showed that LUM expression in 13 datasets (GSE4412, GSE9891, GSE3494, GSE9893, GSE17536, GSE4475, GSE13507, GSE4271, MGH-glioma, E-DKFZ-1, GSE7849, GSE3143, and GSE17536) was associated with prognosis closely, where LUM was a risk factor in GBM, OV, colorectal cancer, BLCA, LGG, glioma, and KIRC (Supplementary Figures S5A–H), and LUM was a protective factor in DLBC (Supplementary Figure S5I). However, the four cohorts on breast cancer, GSE3494, GSE3143, GSE9893, and GSE7849, presented contradictory results (Supplementary Figures S6A–D). Thus, we performed a meta-analysis of 19 datasets on breast cancer, involving 31 cohorts. As shown in Supplementary Figure S6E, the overall prognostic tendency of LUM in breast cancer was a risk factor, although the results were not statistically different. In general, LUM is a protective gene for DLBC, LIHC, UCEC, as well as UCS, and an adverse factor for ACC, BLCA, GBM, KIRC, KIRP, LGG, PAAD, STAD, TGCT, ESCA, as well as LGG.

### 3.3 Correlations between LUM expression with TME in pan-cancer

We evaluated stromal and immune scores for 33 cancers using the ESTIMATE algorithm and analyzed the correlations between these two scores and LUM. LUM levels were significantly positively correlated with immune scores in 8 tumor types, including BLCA, COAD, CHOL, GBM, PAAD, PCPG, PRAD, and THCA, and stromal scores in 28 tumor types, including BLCA, BRCA, CHOL, CESC, COAD, ESCA, GBM, HNSC, KIRP, KICH, KIRC, LIHC, LUAD, LUSC, LGG, MESO, OV, PCPG, PRAD, PAAD, READ, SARC, STAD, SKCM, TGCT, THCA, UCS, and UCEC (Supplementary Figure S7).

**FIGURE 5:**
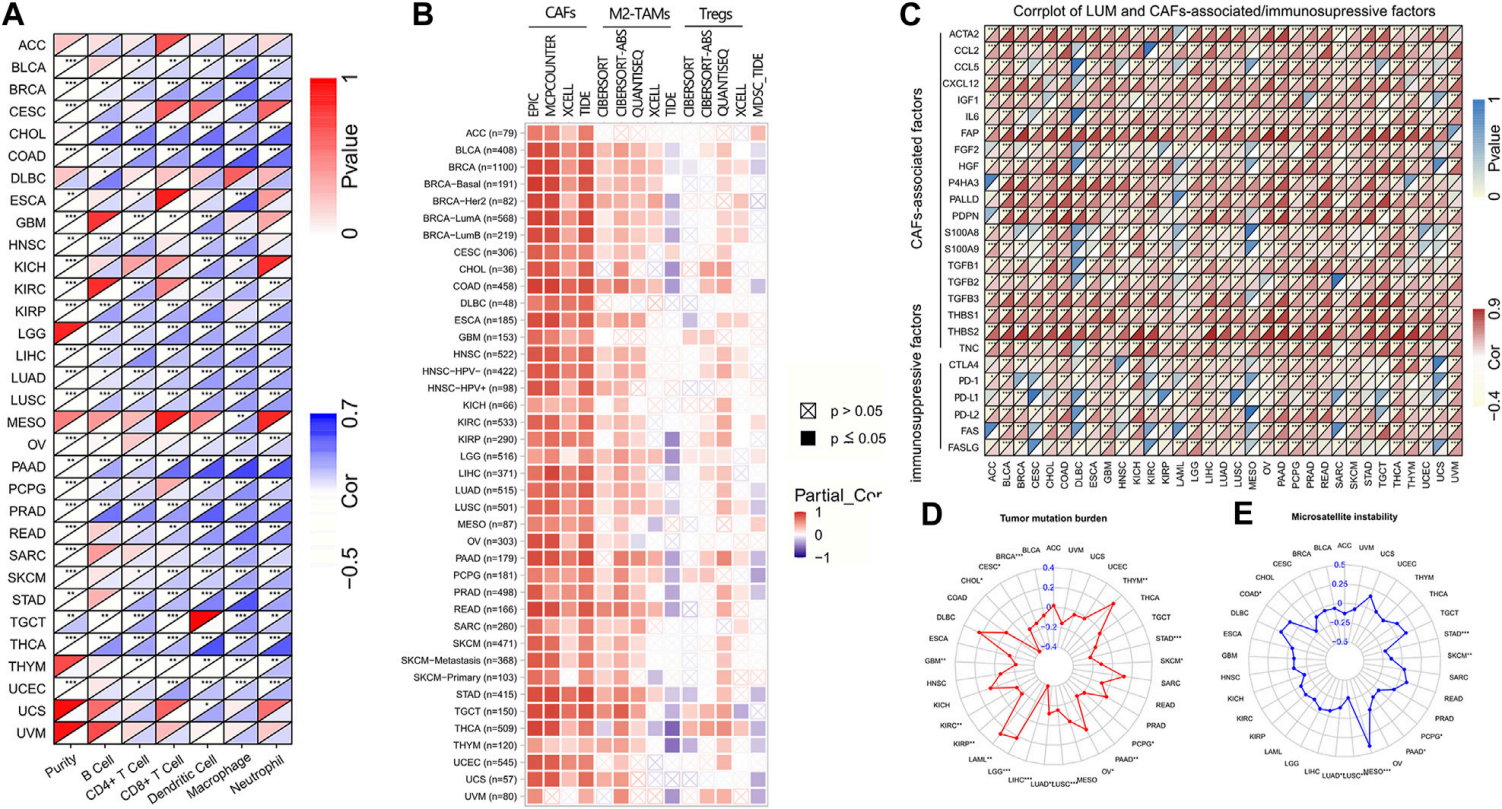
The correlation of LUM expression with immune cells, immunosuppressive cells and factors, TMB, and MSI. Figure **(A,B)** display the heat maps showing the association of LUM expression with 6 immune cell types and 4 immunosuppressive cell types in different TCGA tumor types. Figure **(C)** presents the relationship between LUM expression level and CAFs-associated/immunosuppressive factors. Figure **(D,E)** are the radar maps displaying the correlation between LUM expression and two immune biomarkers (TMB and MSI). (*p < 0.05, **p < 0.01, ***p < 0.001).

Then, we used the TIMER2 database to assess the association between LUM and tumor immune cells. Figure 5A shows that expressions of LUM had a good correlation with immune cells in most tumors, among which BRCA, CHOL, COAD, LGG, LIHC, LUAD, LUSC, PAAD, PRAD, and THCA were positively correlated with all 6 immune cell types.

We also assessed the relationship between infiltrations of 4 immunosuppressive cells and LUM expressions. LUM expressions were positively correlated with CAFs, derived by various algorithms in almost all tumors. Although correlations between LUM and M2-TAMs/Tregs were not completely consistent in different algorithms, LUM expressions were positively correlated with M2-TAMs in BLCA, BRCA, COAD, CESC, ESCA, KIRC, LIHC, LUAD, LUSC, LGG, PCPG, PRAD, PAAD, READ, STAD, and TGCT, and Tregs in BLCA, BRCA, COAD, LUAD, LUSC, PAAD, PCPG, PRAD, STAD, THCA, THYM, UCS, and UVM. Expressions of LUM in a large proportion of tumors, including BLCA, BRCA, COAD, LUAD, LUSC, PAAD, PCPG, PRAD, STAD, THCA, THYM, UCS, and UVM were inversely associated with the degree of MDSCs levels (Figure 5B). Figure 5C shows significant positive correlations between LUM and most CAFs-associated factors, verifying that the function of LUM is closely related to CAFs. Notably, LUM expressions in some tumors were positively correlated with common immune checkpoint genes, such as CTLA4, PD-1, PD-L1, and PD-L2.

Next, correlations between LUM expressions and TMB/MSI were evaluated by a radar map to predict immunotherapeutic efficacies. Expressions of LUM were negatively correlated with TMB of STAD, SKCM, PCPG, PAAD, LUAD, LUSC, LIHC, KIRP, KIRC, GBM, CHOL, CESC, and BRCA, but positively correlated with TMB of THYM, OV, LGG, and LAML (Figure 5D). Expressions of LUM were negatively correlated with MSI of STAD, SKCM, PCPG, PAAD, LUAD, and LUSC, but positively correlated with MSI of MESO and COAD (Figure 5E). There were no overlapping tumors in positive correlations among TMB, MSI and LUM. However, STAD, SKCM, PCPG, PAAD, LUAD, and LUSC all showed negative correlations between LUM and TMB or MSI.

**FIGURE 6:**
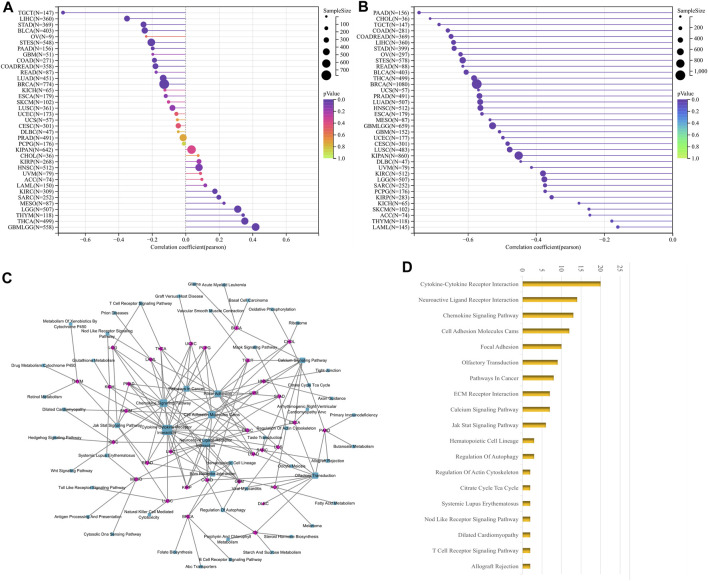
Correlation analysis of LUM with tumor stemness index and GSEA functional enrichment analysis of LUM in pan-cancer. The association between LUM expression and tumor stemness was visualized, including DNAss **(A)** and RNAss **(B)**. The five pathways with the lowest *p*-values regarding LUM for 33 tumors were visualized together **(C)**. The bar chart shows the pathways with degree values greater than 1 **(D)**.

DNAss reflects epigenetic characteristics and RNAss reflects gene expression. The higher these two tumor stem cell indices, the more deficient the immune cell infiltration in the tumor microenvironment and the lower the PD-L1 expression (Malta et al., 2018). The findings showed that LUM expression, negatively correlated with DNAss in 10 tumor types, including STAD, LIHC and TGCT; positively correlated with DNAss in 8 tumor types, including LGG, THYM and THCA (Figure 6A); and significantly negatively correlated with RNAss in all tumor types (Figure 6B).

We then carried out GSEA analysis to investigate potential LUM involvement in tumor pathways. For each tumor, the 5 pathways with the lowest *p*-value were displayed (Supplementary Table S1). LUM was primarily involved in processes like signaling and cell adhesion, such as Cytokine-Cytokine Receptor Interaction and Cell Adhesion Molecules, as shown in Figures 6C, D.

### 3.4 Patients with elevated LUM levels were sensitive to chemotherapy, not immunotherapy

**FIGURE 7:**
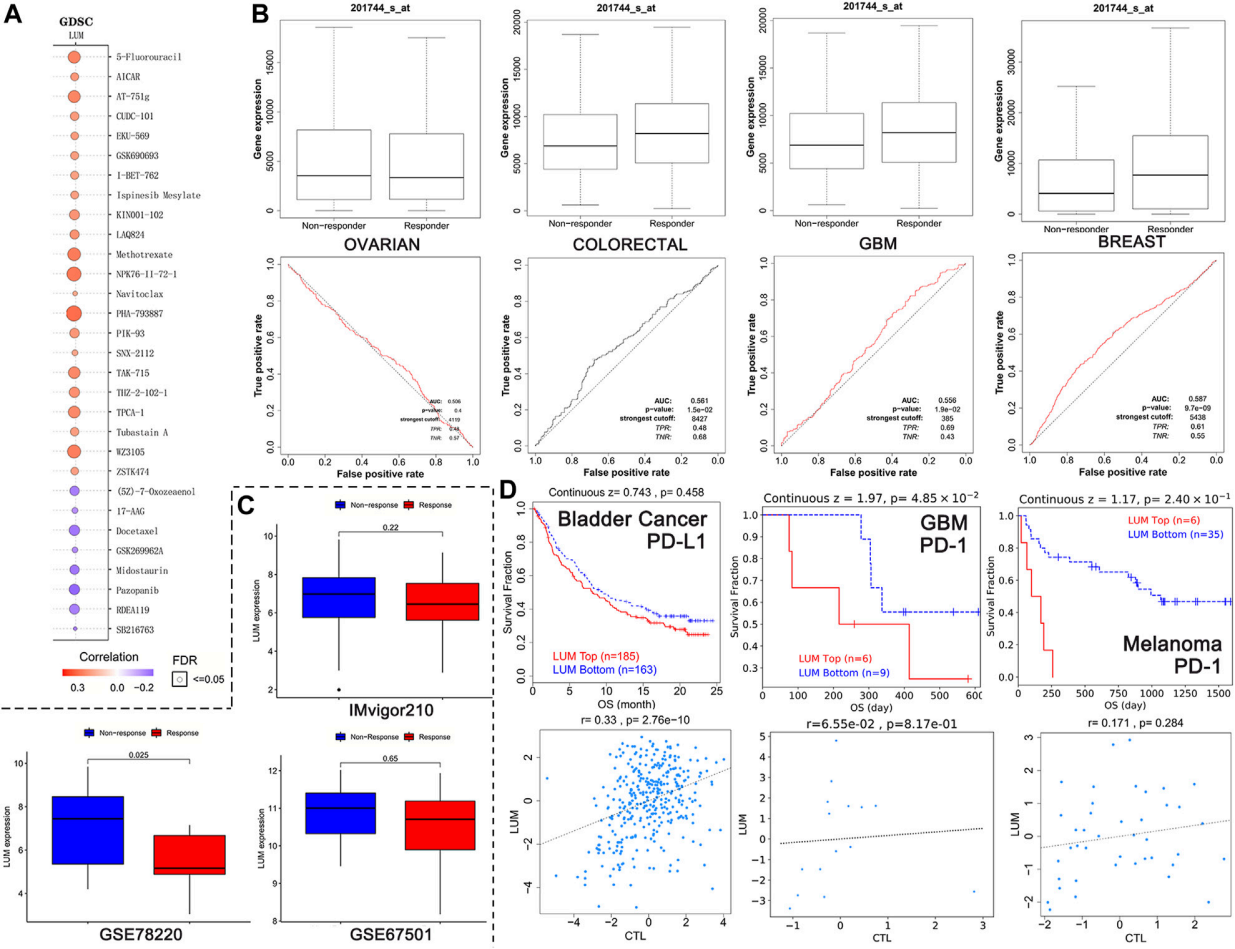
Relationship between LUM expression and treatment response. **(A)** the 30 drugs most associated with LUM expression in the GDSC database. The redder the circle, the more resistant cells with high LUM expression are to the drug. The size of the circle represents false detection Rate (FDR). The smaller the FDR, the more reliable the results are. **(B)** the ROC curves showing the association between chemotherapy response and LUM expression in OV, colorectal cancer, GBM and breast cancer cohorts. **(C)** LUM expression tended to be higher in the immunotherapy non-responder group than in the immunotherapy responder group. **(D)** Kaplan-Meier analysis showing differences in response to immunotherapy between the LUM high expression group and the LUM low expression group. A simple linear regression model showing the correlation between LUM expression and CTL in the indicated cohorts.

The relationship between LUM levels and GDSC drug sensitivity was evaluated using the GSCA website. Elevated LUM levels were correlated with increased sensitivity of cancer cell lines to (5Z)-7-Oxozeaenol, 17-AAG, docetaxel, GSK269962A, midostaurin, pazopanib, RDEA119, as well as SB216763 and decreased activities of 5-Fluorouracil, AICAR, AT-751g, CUDC-101, EKU-569, GSK690693, I-BET-762, ispinesib mesylate, KIN001-102, LAQ824, methotrexate, NPK76-II-72-1, navitoclax, PHA-793887, PIK-93, SNX-2112, TAK-715, THZ-2-102-1, TPCA-1, tubastain A, WZ3105, and ZSTK474 in various cancer cell lines (Figure 7A).

The impact of LUM on chemotherapeutic responses in different tumor cohorts was also determined. It was found that OV patients with elevated LUM levels were not sensitive to chemotherapy, while breast cancer, colorectal cancer and GBM patients with elevated LUM levels had greater chemotherapeutic benefits, relative to those with low expressions (Figure 7B).

The relationships between LUM expressions and immunotherapeutic responses were validated by three independent cohorts (Figure 7C). There were no significant differences between LUM and immunotherapeutic responses in GSE67501 cohort with renal cell carcinoma and IMvigor210 cohort with advanced urothelial cancer. However, in the GSE78220 cohort with melanoma, we found that high LUM expression was more likely to be unresponsive to immunotherapy, which is consistent with TMB and MSI predictions.

To investigate the reasons for poor immunotherapeutic effects in patients with high LUM expressions, a LUM gene query was conducted on the TIDE website. In bladder cancer, GBM and melanoma, relations between LUM and prognosis and the relation between LUM and CTL were inconsistent (Figure 7D). The higher the CTL level, the better the patient’s prognosis.

## 4 Discussion

LUM is involved in tumorigenesis and progression, including cell transformation, hyperplasia, adhesion and invasion (Nikitovic et al., 2011; Brézillon et al., 2013; Karamanou et al., 2020). In most tumors, differentially expressed LUM have been observed in cancer and normal tissues. Findings on the role of LUM in most cancer types are contradictory (Simões et al., 2017). Over-expressed LUM has been found in various cancers, including BRCA, COAD, GBM, HNSC, PAAD, and STAD, in accordance with previous findings. Previous studies (Li et al., 2014; Zhang et al., 2016) reported that LUM is the most abundant protein polysaccharide in BRCA, and LUM expressions in cancer cells are high. Wang et al. reported that LUM expressions are strongly upregulated in STAD (Wang et al., 2017). In partially differentiated non-proliferating cells, LUM expressions are negative or weak. In 16 cancer types (LIHC, LUAD, KIRC, OV, and UCEC), we found suppressed LUM levels in tumor tissues, relative to neighboring non-tumor tissues. In OVA, LUM was found to be transcriptionally repressed by the HMGA2 oncogene (Wu et al., 2011). Furthermore, LUM was established to be weakly expressed in UCEC, and LUM expressions negatively correlated with UCEC invasion and metastasis (Wu et al., 2020). These differences may have been due to different mutation frequencies and types of LUM in different tumor types, and experimental verification is required.

LUM expressions have been reported to be higher in metastatic gastric, colon, and renal clear cancers, relative to non-metastatic groups. Moreover, by inhibiting FOXO3 and weakening its binding with the LUM promoter, LUM expressions are suppressed, leading to reduced migration of neuroblastoma cells (Salcher et al., 2019). Our diffuse analysis revealed that LUM levels are elevated in advanced BLCA, ESCA, KIRC, OV, and THCA patients. Moreover, LUM protein levels are highly elevated in gastric cancer (Chen et al., 2017), colon cancer (Seya et al., 2006; de Wit et al., 2013) and pancreatic cancer (Li et al., 2014). In general, protein expressions reflect LUM activities. Currently, there is no database of protein expression levels in all tumors. However, in this study, based on CPTAC data, LUM levels were established to be suppressed in BRCA, COAD, HNSC, LUAD, LIHC, OV, as well as UCEC and elevated in GBM, KIRC and PAAD. Moreover, the results of immunohistochemistry of BRCA, COAD, HNSC, KIRC, and PRAD were inconsistent with the results of genomics and proteomics, and we have not been able to explore the reasons for this in depth due to the fact that very little research has been done on LUM, including methylation and protein phosphorylation. We hypothesize that this inconsistency is the result of statistical errors or changes in the transcription process. Examples include amplification of the LUM gene or deletions at the locus, such as the E240K deletion. Several studies have identified an important role for E240K in antibiotics (Jones et al., 2009), and we expect that studies will be conducted to explore the relationship between E240K, LUM, and tumors in greater depth. In conclusion, we found that PAAD was the only tumor in our study with consistently elevated LUM expression in all three analyses: RNA analysis, protein analysis, and immunohistochemical analysis.

With regards to growth and metastasis, LUM plays a dual regulatory role in several cancer types, however, the specific mechanisms have yet to be established (Li et al., 2014). The possible mechanisms may depend on tumor type, cellular context, and disease stage. In terms of survival, KM and Cox regression analyses showed that LUM expressions were protective in DLBC, LIHC, UCEC, and UCS, whereas, it seemed to be a risk element in ACC, BLCA, GBM, KIRC, KIRP, LGG, PAAD, STAD, TGCT, ESCA, and LGG. Elevated LUM levels have been correlated with unfavorable clinical outcomes (Chen et al., 2017). Mao et al. observed that bladder cancer patients with elevated LUM expressions correlated with poorer OS, relative to patients with suppressed LUM expressions (Appunni et al., 2021). In PAAD, over-expressed LUM is indicative of the late TNM stage, and is associated with poor OS outcomes (Song et al., 2021). Moreover, in STAD, LUM expressions were closely associated with metastatic dissemination, lymphatic metastasis, and poor prognostic outcomes (Sheppard et al., 2018). We established that LUM expressions negatively correlated with long-term survival outcomes of STAD patients, implying that LUM functions as an oncogene in STAD. In ESCA patients, elevated LUM levels suggest poor outcomes (Yamauchi et al., 2021). The above results suggest that elevated LUM expressions predict poor prognostic outcomes for these cancer patients. On the contrary, LUM expressions have been suggested to attenuate tumor progression, which is associated with favorable clinical outcomes (Sarvaria et al., 2017). In DLBC, LIHC, UCEC, and UCS, we established that LUM overexpressions predicted good survival outcomes, implying that LUM has anti-tumorigenic effects.

In this study, eight tumor types showed good associations between LUM expressions and immune scores. Stromal cells are an essential part of TME and have important functions in cancer biology. LUM expressions were positively associated with stromal scores in most tumors. The eight cancer types that were positively correlated with immune scores exhibited good overall positive correlations with infiltration levels of six immune cell types. Assessment of the association between LUM expressions and four immunosuppressive cells in 33 human cancers revealed that LUM expressions were positively correlated with CAFs in almost all tumors. To our knowledge, tumors usually present as connective tissue hyperplasia that increases deposition and cross binding of ECM proteins, with CAFs, as the main ECM producers, being the dominant cell type. In a previous study, gastric cancer cells co-cultured with LUM knockout CAFs exhibited significantly reduced proliferation and metastasis *in vivo*. Therefore, we postulate that the LUM protein is mostly produced by CAFs and may be one of the causes of disease deterioration in tumor patients.

Additionally, we investigated the association between LUM and two immunotherapeutic biomarkers. TMB and MSI, measured by comprehensive genomic profiling, are reliable predictors of immunotherapeutic responses (Cancer Genome Atlas Research Network, 2014; Johnson et al., 2016). Patients with TMB-high or MSI-high tumors exhibited perfect clinical responses to PD-1/PD-L1 blockade (Goodman et al., 2017). In this study, LUM was inversely correlated with TMB and MSI in SKCM, STAD, PCPG, PAAD, LUAD and LUSC. These findings suggest that patients with elevated LUM expressions have poor clinical responses to immunotherapy.

To inform the clinical applications of LUM, we evaluated the correlations between LUM with chemotherapy and immunotherapy. In various cancer cell lines, elevated LUM expressions were associated with decreased sensitivity to AICAR (an AMPK activator). Autophagy is a type of protective cellular response to chemotherapies (Li et al., 2016). LUM suppressed AMPK activities and inhibited chemotherapeutic agent-induced autophagy, thereby augmenting the cytotoxicity of chemotherapy. This further supports our findings. Furthermore, we determined that higher LUM levels enhanced 17-AAG activities, which is a selective inhibitor of HSP90 (Talaei et al., 2019). LUM has been shown to be associated with HSP90 and a lack of LUM expressions in human breast cancer may limit the clinical efficacies of HSP90 inhibitors (Shipp et al., 2011). LUM expressions were cancer type-dependently differentially associated with chemotherapeutic responses. We observed that colorectal, GBM, and breast cancer patients with high LUM expressions have good clinical responses to chemotherapy, while ovarian cancer patients with high LUM expressions are resistant to chemotherapies. Because our study explored the prediction of LUM with immunotherapy and chemotherapy, this combined approach has not found anyone who has done the same analysis, so it cannot find similar genes. However, regarding immune efficacy, we found that N6AMT1 had the same immunotherapy predictive effect as LUM (Wang et al., 2023), while SLC35A2 had the opposite immunotherapy predictive effect as LUM (Xu et al., 2023). Therefore, the development of targeted drugs that inhibit LUM expression and thus promote the efficacy of immunotherapy is also a worthwhile way to try.

We have observed a trend that patients with high LUM levels tend to be more insensitive to immunotherapy. In addition, we found that three cohorts of bladder cancer, glioblastoma, and melanoma with low LUM expressions had higher survival outcomes after ICB treatment, confirming the importance of low LUM levels in immunotherapy. Surprisingly, elevated levels of LUM expressions in these queues were positively associated with CTL. CTLs are important in elimination of malignant cells. However, CTL-mediated tumor killing exhibit a long-lasting clinical efficacy in extremely few cancer patients (Hope et al., 2019). The small success rates may be attributed to T cell exhaustion, which involves abnormalities of TME. Continuous challenge with chronic antigens can progressively cause T cell exhaustion, as T cells lose the ability to proliferate, produce key cytokines, and kill target cells, ultimately preventing optimal antitumor T-cell responses (Miao et al., 2017). CAFs have been shown to upregulate the expressions of immune checkpoint molecules, such as CTLA4/B7, PD-1/PD-L, PD-1/PD-L2, and FAS/FASL, thereby inducing T cell dysfunctions (Lakins et al., 2018; Mao et al., 2021). In this study, LUM was strongly associated with high expression levels of immunosuppressive receptors (PD-1, PD-L2, etc.), which are characteristics of T cell exhaustion or dysfunction (Franco et al., 2020). Therefore, we infer that LUM is closely related to the TME, where immunosuppressive factors and T cell dysfunction play a significant role.

## 5 Conclusion

Our findings imply that LUM is a potential prognostic factor for cancers. Its expression is significantly associated with TME, therapeutic responses, and pathogenesis. Among them, LUM was positively correlated with CAFs, and high expression of LUM showed poor efficacy in the immunotherapy cohort, further confirming the involvement of lumican in immunosuppression. Identifying high-risk patients through LUM and formulating personalized treatment plans will hopefully improve their prognosis. The results in this study should be verified via *in vivo* and *in vitro* experiments.

## Data Availability

The original contributions presented in the study are included in the article/Supplementary Material, further inquiries can be directed to the corresponding author.

## Supplementary material

SUPPLEMENTARY TABLE S1 GSEA functional enrichment analysis of LUM from 33 tumors.
