# Supplementary figures and images for: Multi-omics analysis reveals the prognostic and tumor micro-environmental value of lumican in multiple cancer types
Source: Front Mol Biosci. 2023 Aug 24;10:1158747. doi: 10.3389/fmolb.2023.1158747 (PMC10484533; doi:10.3389/fmolb.2023.1158747)

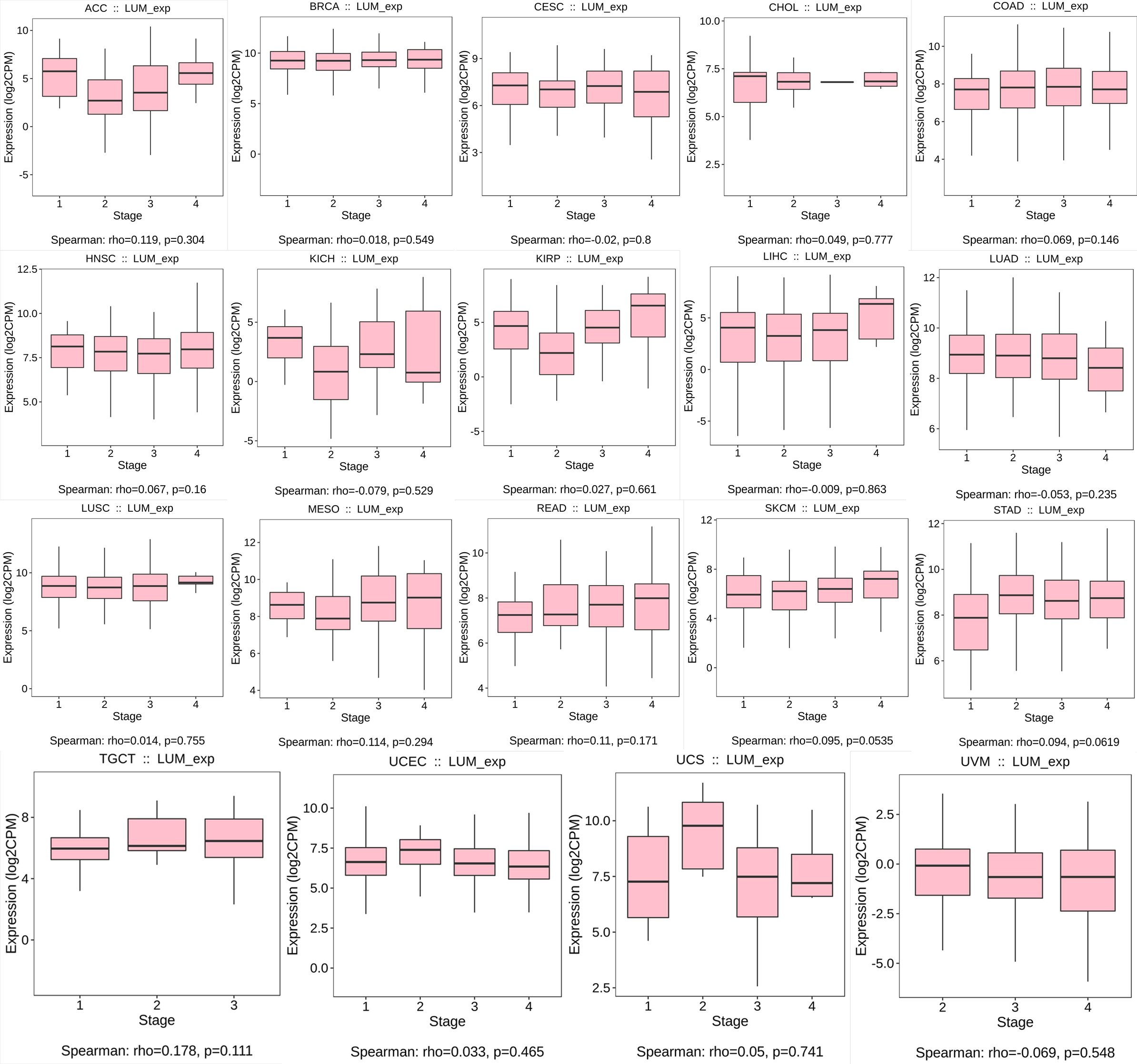

Supplement: Supplementary file 1 [file Image3.TIF]

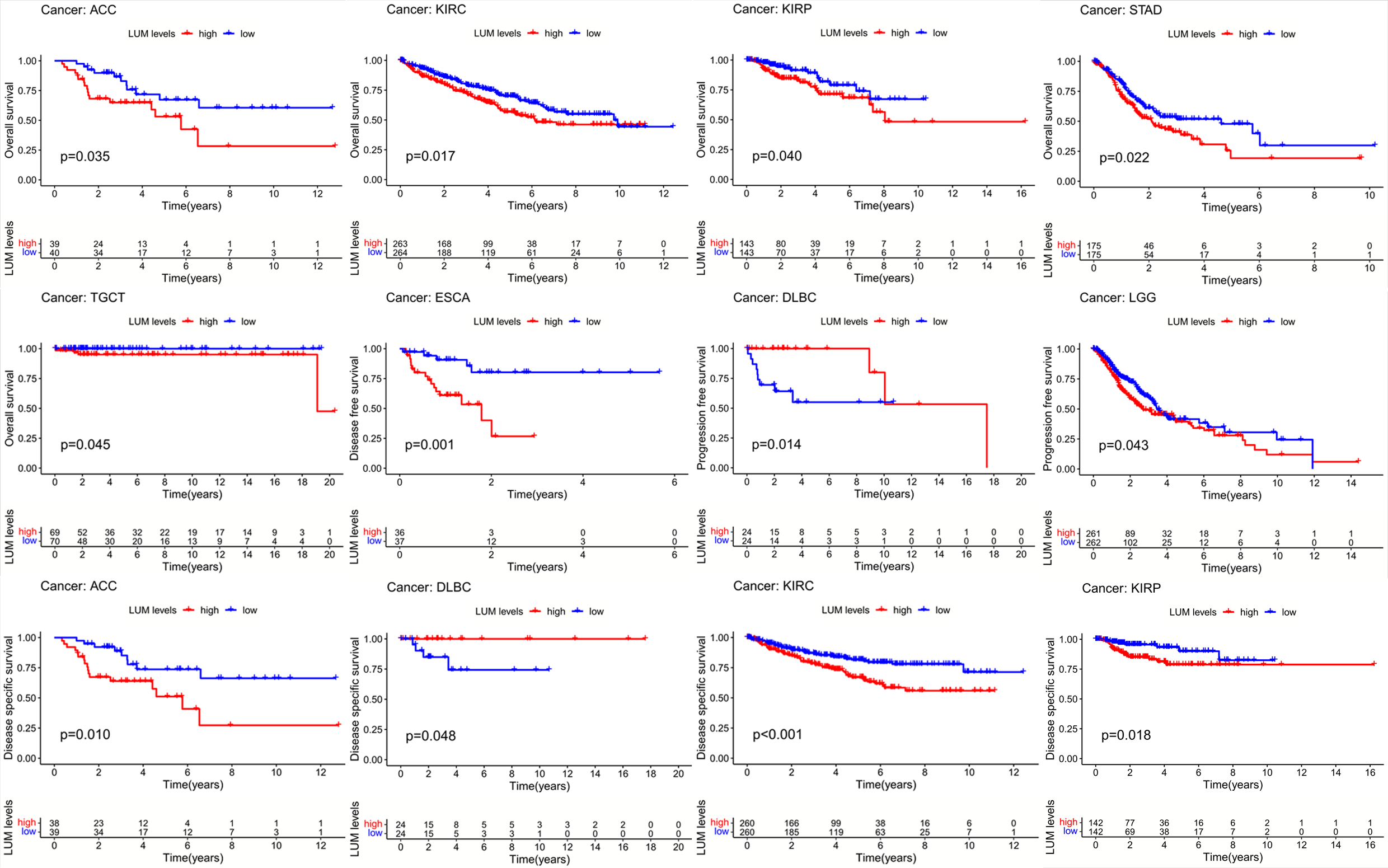

Supplement: Supplementary file 2 [file Image4.TIF]

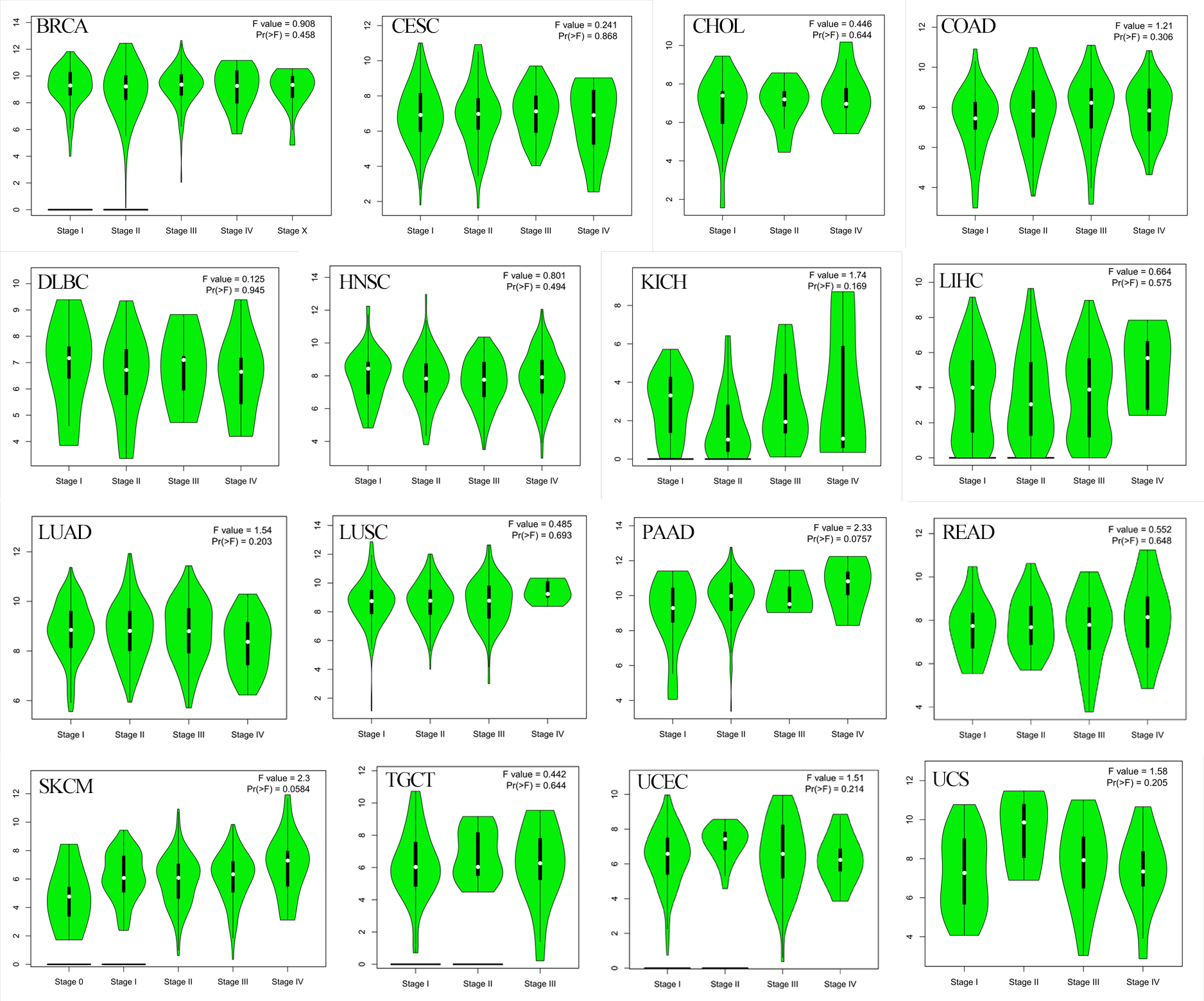

Supplement: Supplementary file 3 [file Image2.TIF]

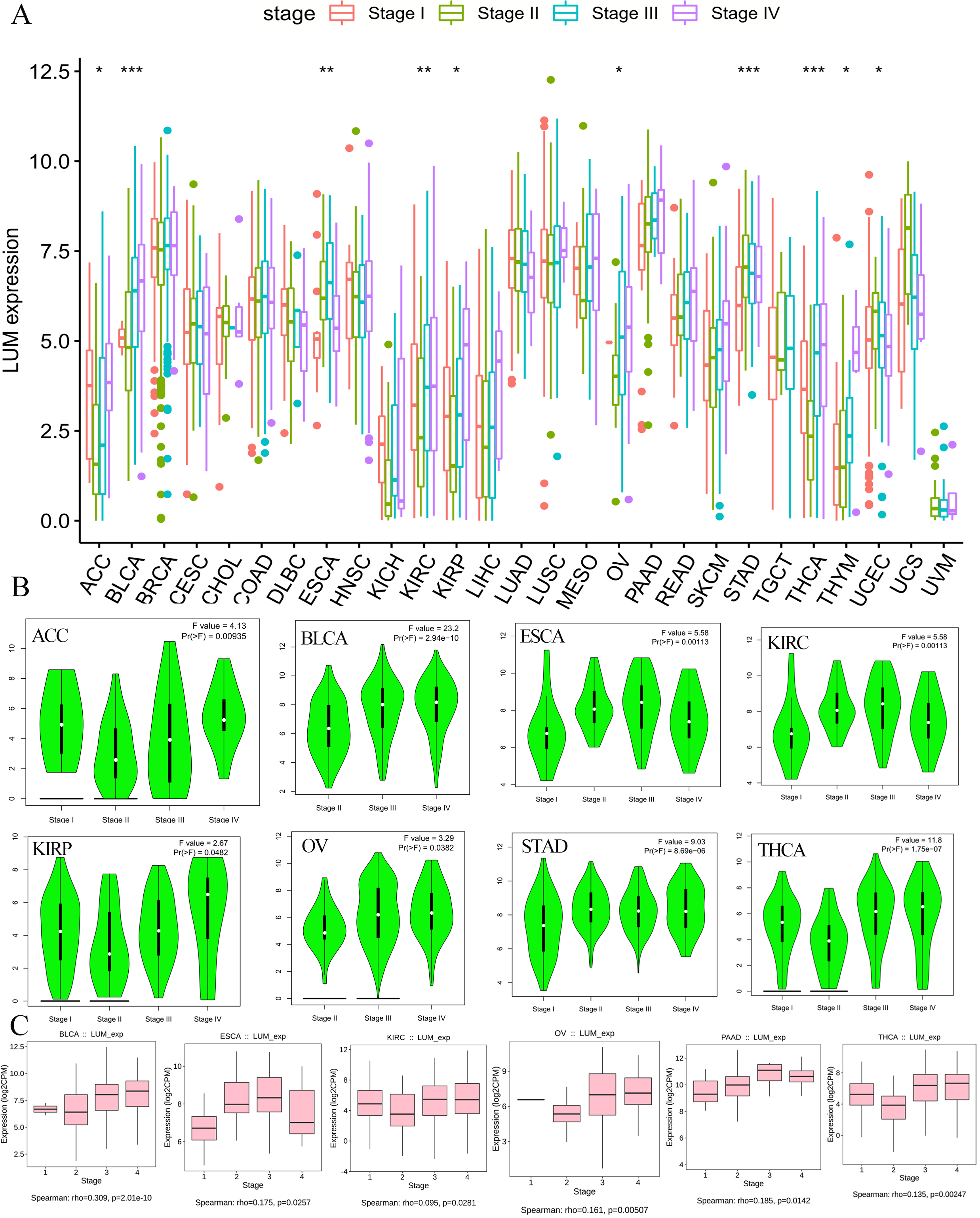

Supplement: Supplementary file 4 [file Image1.TIF]

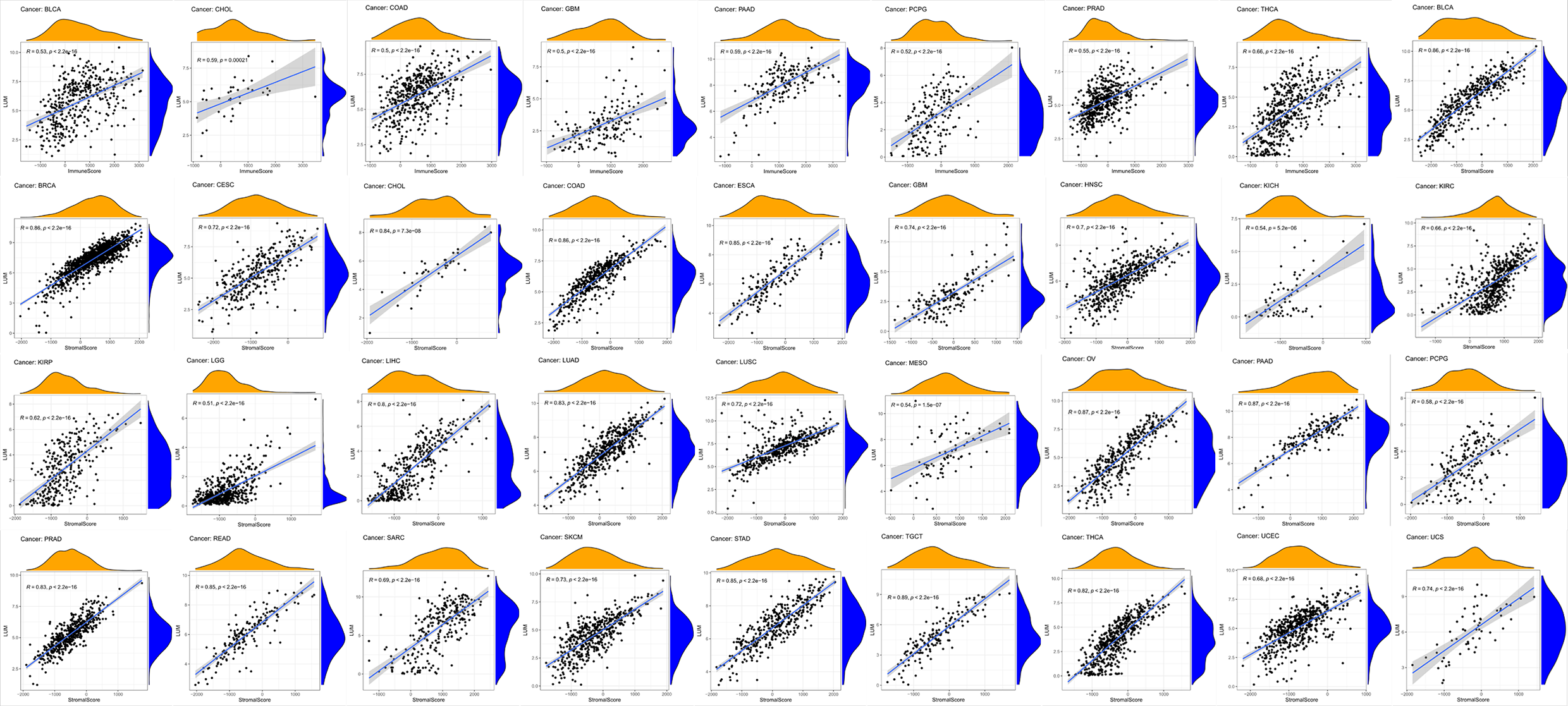

Supplement: Supplementary file 5 [file Image7.TIF]

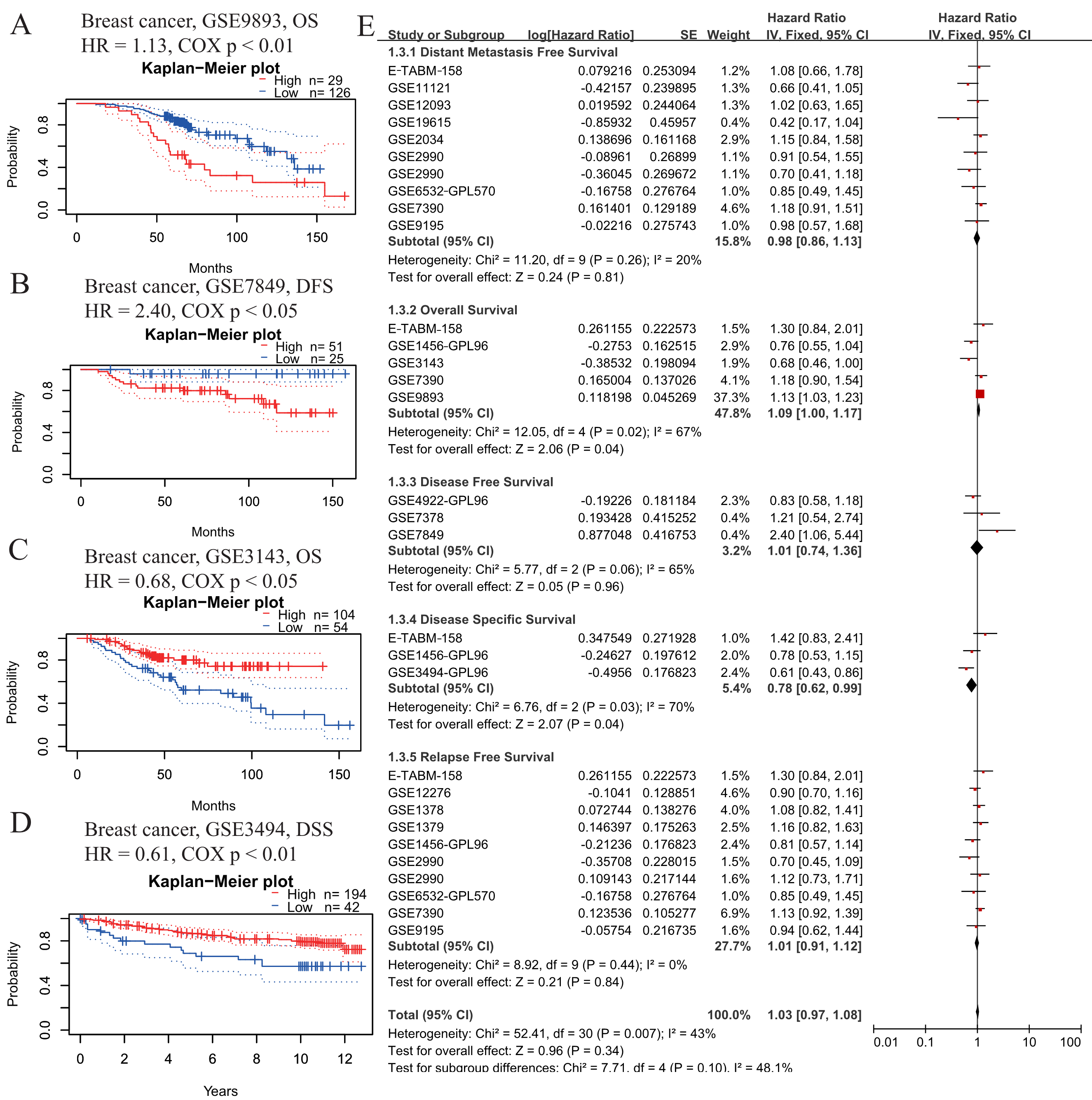

Supplement: Supplementary file 6 [file Image6.TIFF]

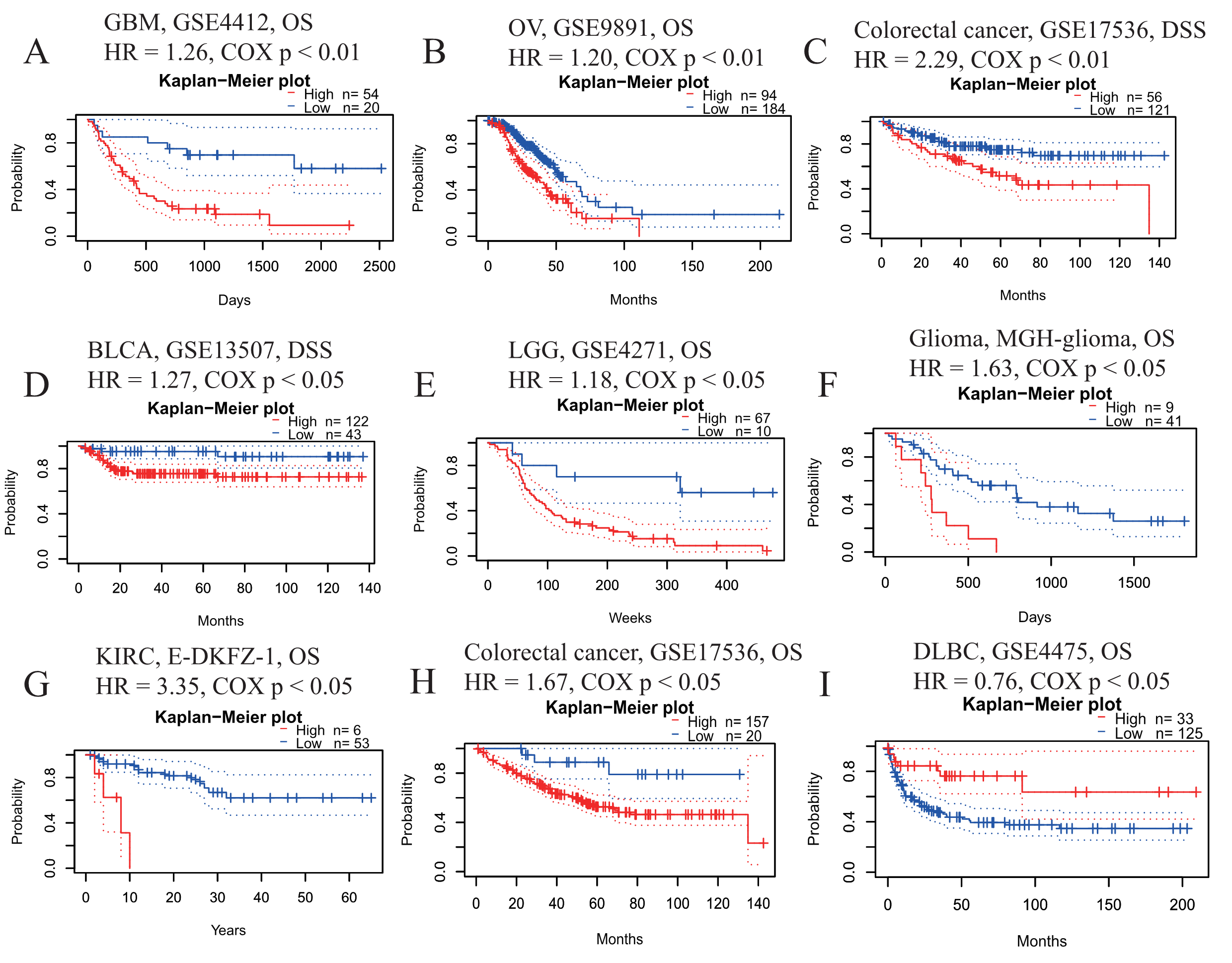

Supplement: Supplementary file 7 [file Image5.TIF]
